# Living with COVID-19: mass gatherings and minimizing risk

**DOI:** 10.1093/qjmed/hcab163

**Published:** 2021-06-09

**Authors:** M Murakami, T Yasutaka, M Onishi, W Naito, N Shinohara, T Okuda, K Fujii, K Katayama, S Imoto

**Affiliations:** 1 Department of Health Risk Communication, Fukushima Medical University School of Medicine, 1 Hikarigaoka, Fukushima, Fukushima 960-1295, Japan; 2 Institute for Geo-Resources and Environment, National Institute of Advanced Industrial Science and Technology (AIST), 1-1-1, Higashi, Tsukuba, Ibaraki 305-8567, Japan; 3 Artificial Intelligence Research Center, National Institute of Advanced Industrial Science and Technology (AIST), 2-4-7 Aomi, Koto-ku, Tokyo 135-0064, Japan; 4 Research Institute of Science for Safety and Sustainability, National Institute of Advanced Industrial Science and Technology (AIST), 16-1, Onogawa, Tsukuba, Ibaraki 305-8569, Japan; 5 Department of Applied Chemistry, Faculty of Science and Technology, Keio University, 3-14-1 Hiyoshi, Kohoku, Yokohama, Kanagawa 223-8522, Japan; 6 R&D-Hygiene Science Research Center, Kao Corporation, 2-1-3, Bunka, Sumida-ku, Tokyo 131-8501, Japan; 7 Laboratory of Sequence Analysis, Human Genome Center, The Institute of Medical Science, The University of Tokyo, 4-6-1 Shirokanedai, Minato-ku, Tokyo 108-8639, Japan; 8 Division of Health Medical Intelligence, Human Genome Center, The Institute of Medical Science, The University of Tokyo, 4-6-1 Shirokanedai, Minato-ku, Tokyo 108-8639, Japan

## Abstract

During the COVID-19 pandemic, it has been important to both minimize the risk of infection and restore daily life. As a typical example, mass gathering events, such as sporting events, are gradually becoming more common, thanks to the measures taken to contain COVID-19. Some pilot studies have been launched at governments’ initiative to investigate the risk of infection without measures such as face masks and physical distancing at mass gathering events, but the ethics of these studies should be carefully considered. On the other hand, it is still beneficial to implement infection control measures at mass gathering events and, in parallel, to estimate the risk of infection with measures in place, especially under a lack of vaccination progress or the spread of mutant strains possibly resistant to vaccines. To help improve compliance with measures taken by spectators and organizers and to ensure their effectiveness, we have conducted quantitative evaluations of the implementation of such measures by monitoring CO_2_ concentrations, assessing the proportion of people wearing face masks and analysing human flow at the event. This approach allows us to share our observations with stakeholders and participants, enabling us to protect the culture of mass gathering events, minimize the risk of infection and restore a sense of well-being in daily life.

Various measures were taken to stop the spread of COVID-19, including lockdowns, but daily life must be gradually restored while continuing to minimize the risk of infection.

Mass gathering events, such as sporting events, are an opportunity to restore a sense of normalcy. While mass gathering events are a pathway of infection, they are also essential for bringing joy into daily life. Mass gathering events, which were restricted during the pandemic, are gradually becoming more common, thanks to the measures taken to contain COVID-19. In the UK, the Events Research Programme was launched at the government’s initiative with the progress of the vaccination programme.^1^ Pilot studies have been conducted to prospectively assess the risk of infection by polymerase chain reaction testing in participants who attend mass gathering events. The risk of infection will also be investigated in combination with the suspension of the use of face masks and physical distancing during mass gathering events. In the Netherlands, where most people remain unvaccinated, the government and researchers are collaborating to conduct empirical pilot studies alongside the use of infection risk models at mass gathering events without face masks.^2^ These approaches provide perspectives to governments by verifying the effectiveness of measures, which can help create a road map for holding mass gathering events. The ethics of these approaches, however, should be carefully considered to ensure good science.^2^

It is still important and beneficial to implement infection control measures at mass gathering events and, in parallel, to estimate the risk of infection with these measures in place and to follow up on the subsequent infection situation, especially given a lack of progress in vaccination and the spread of mutant strains possibly resistant to vaccines. The infection routes of COVID-19 include droplet transmission (direct exposure to the virus in the vicinity of infectors), airborne transmission (exposure caused by inhalation of the virus in the air) and contact transmission (exposure from hand-touch contact with the virus deposited on environmental surfaces). It has been estimated by quantitative microbial risk assessment that the implementation of measures according to the route of infection, such as physical distancing and wearing of face masks for droplet transmission, ventilation for airborne transmission and decontamination and hand washing for contact transmission, is useful for controlling the risk of infection at mass gathering events.^3^ Therefore, it is important to comply with the implemented measures and to ensure the effective compliance of spectators, who must wear face masks and maintain physical distancing, as well as organizers, who are responsible for ventilation and decontamination of the venue.

We have conducted quantitative evaluations of the implementation of these measures by monitoring CO_2_ concentrations, assessing the proportion of people wearing face masks and analysing human flows at professional events, such as sports games, leading to the improvement of the measures.^4^^,^^5^ CO_2_ measurements can be used to monitor human crowding and ventilation (Figure 1a). In practice, high CO_2_ concentrations were observed when people gathered in restrooms during a break in events; in subsequent events, real-time CO_2_ observation was implemented in several stadiums to reduce human crowding. The proportion of people wearing face masks can be estimated without collecting personal information by analysing video footage with artificial intelligence (Figure 1b). The average proportion of people wearing face masks at the events studied was more than 90%,^5^ confirming high adherence. Human flow analysis, measured by laser imaging detection and ranging sensors, enables the evaluation of the extent to which human crowding is reduced by dispersed entry and exit (Figure 1c).

**Figure 1.**
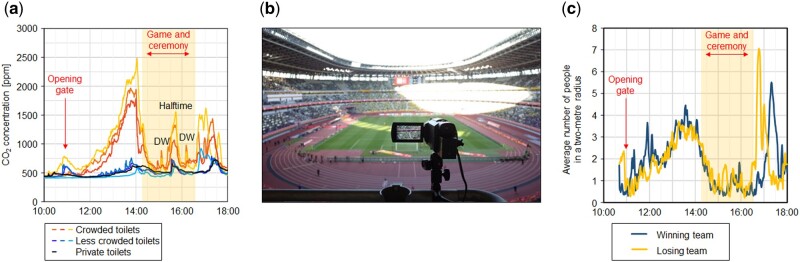
Investigation of the implementation of public health measures in the Japan National Stadium at J. League YBC Levain CUP held on 4 January 2021. (**a**) CO_2_ concentrations. (**b**) Analysis of the proportion of people wearing face masks using video with artificial intelligence. (**c**) Human flow analysis. The event was held with 24 219 spectators, which is less than a limited capacity of 50%. Figures (a–c) were created with modifications based on refs.^4^^,^^5^ DW, time for drinking water.

Sharing these observations and the effects of public health measures with participants will be essential to controlling the risk of COVID-19 and preserving the culture of mass gathering events. For example, real-time feedback on the proportion of people wearing face masks can be expected to help participants maintain high adherence, hopefully without any psychological burden and with enjoyment. The culture of mass gathering events is fostered not only by athletes, artists and their associates but also by fans. Understanding and mitigating the health risks of mass gathering events will protect the culture, minimize the risk of infection and restore a sense of well-being in daily life.

## Funding

No external financial support is used for this article.

*Conflict of interest.* This research project comprises other members from two private companies, Kao Corporation and NVIDIA Corporation, Japan. K.F. is affiliated with Kao Corporation. W.N. received financial support from the Kao Corporation until March 2020 in context outside the submitted work. T.Y., M.O., W.N., N.S. and T.O. have received financial support from the Kao Corporation for a collaborative research project in the context of measures at mass gathering events. T.Y., M.O., W.N. and N.S. have received financial support from Yomiuri Giants, Japan Professional Football League and Japan Professional Basketball League. M.M., T.Y., M.O., N.S., W.N. and S.I. attend the new coronavirus countermeasures liaison council jointly established by the Nippon Professional Baseball Organization and Japan Professional Football League as experts without any rewards. T.Y., M.O., W.N., N.S. and K.F. are advisors to the Japan National Stadium. Other authors declare no competing interests. The findings and conclusions of this article are solely the responsibility of the authors and do not represent the official views of any institution.
